# The sequential management of recurrent temporomandibular joint ankylosis in a growing child: a case report

**DOI:** 10.1186/s40902-016-0083-z

**Published:** 2016-10-05

**Authors:** Jung-Won Cho, Jung-Hyun Park, Jin-Woo Kim, Sun-Jong Kim

**Affiliations:** Department of Oral and Maxillofacial Surgery, Ewha Womans University Medical Center, 1071, Anyangcheon-ro, Yangchen-gu, Seoul, 07985 South Korea

**Keywords:** TMJ ankylosis, Recurrent ankylosis, Pediatric patient TMJ ankylosis

## Abstract

**Background:**

Temporomandibular joint (TMJ) ankylosis in children often leads to facial deformity, functional deficit, and negative influence of the psychosocial development, which worsens with growth. The treatment of TMJ ankylosis in the pediatric patient is much more challenging than in adults because of a high incidence of recurrence and unfavorable growth of the mandible.

**Case report:**

This is a case report describing sequential management of the left TMJ ankylosis resulted from trauma in early childhood. The multiple surgeries including a costochondral graft and gap arthroplasty using interpositional silicone block were performed, but re-ankylosis of the TMJ occurred after surgery. Alloplastic TMJ prosthesis was conducted to prevent another ankylosis, and signs or symptoms of re-ankylosis were not found. Additional reconstruction surgery was performed to compensate mandibular growth after confirming growth completion. During the first 3 years of long-term follow-up, satisfactory functional and esthetic results were observed.

**Conclusions:**

This is to review the sequential management for the recurrent TMJ ankylosis in a growing child. Even though proper healing was expected after reconstruction of the left TMJ with costal cartilage graft, additional surgical interventions, including interpositional arthroplasty, were performed due to re-ankylosis of the affected site. In this case, alloplastic prosthesis could be an option to prevent TMJ re-ankylosis for growing pediatric patients with TMJ ankylosis in the beginning.

## Background

Temporomandibular joint (TMJ) ankylosis can be defined as the union of mandibular condyle to the cranial base which is the articular surface through osseous or fibrous tissue, with partial or complete mandibular impediment [1]. The etiological factors for TMJ ankylosis include trauma, rheumatoid arthritis, congenital anomalies, infection, and neoplastic processes. Trauma is well known as the most predominant factor in TMJ ankylosis particularly in children and is associated with inadvertent use of forceps during delivery, traffic accident, and falls [2–4]. When TMJ ankylosis occurs in children, the future growth and development of the jaws and teeth are affected negatively. Furthermore, psychosocial development of the children affected is profoundly influenced due to the obvious facial deformity, which worsens as they grow [5]. Condyle reconstruction is carried out in order to restore TMJ function and facial deformity in adults, whereas high incidence of recurrence and the probable change in the unfavorable growth of the mandible are also needed to be considered in children [5, 6].

It is generally recommended that as soon as the condition is diagnosed, the surgery of TMJ ankylosis should be initiated. The main purpose of the surgery is the re-establishment of joint and harmonious jaw functions in children [5, 7]. This case report presents sequential management of recurrent TMJ ankylosis with a variety of methods in a growing child.

## Case presentation

**Fig. 1:**
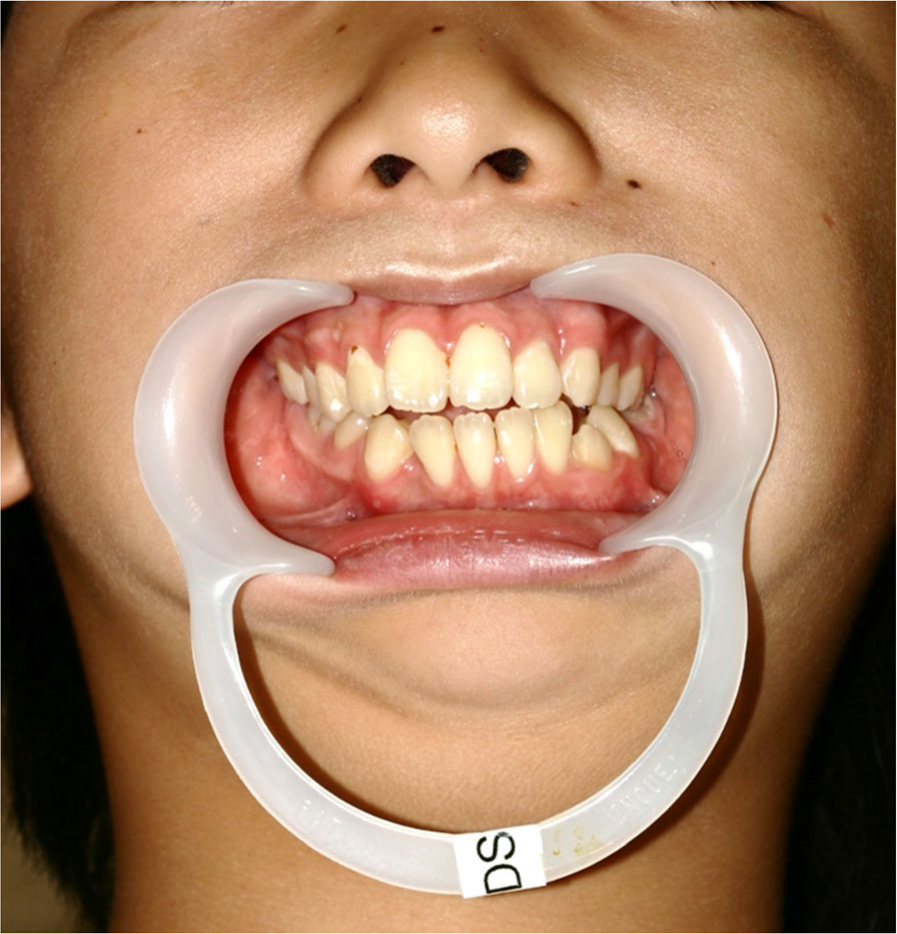
Frontal view after the formation of re-ankylosis when he was 12 years old

**Fig. 2:**
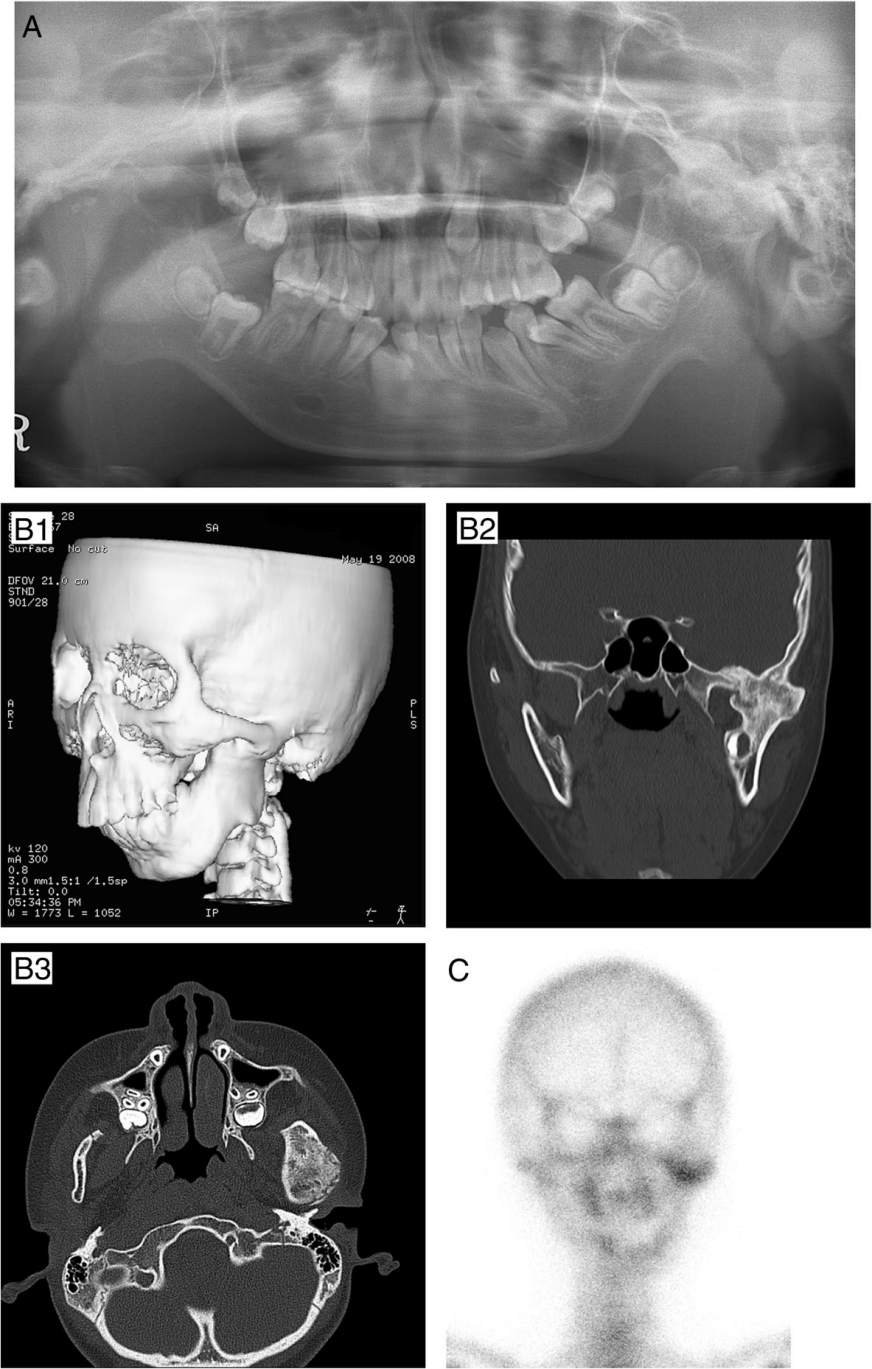
Initial radiographic evaluation. **a** Preoperative panoramic radiograph. Bone mass of the left mandibular condylar process. **b** Preoperative three-dimensional computed tomography. Irregular expanded bony contour, cortical thickening, and diffused sclerosis around the left TMJ. **c** Preoperative three-dimensional computed tomography. Irregular expanded bony contour, cortical thickening, and diffused sclerosis around the left TMJ

A 12-year-old male patient was referred to the Department of Oral and Maxillofacial Surgery at Ewha Womans University Mokdong Hospital for evaluation and treatment of left TMJ ankylosis. He had been diagnosed with bony ankylosis of the left TMJ due to trauma at the age of 1. At 8 years of age, the patient had received TMJ gap arthroplasty with condylectomy at a different hospital. However, he had a difficulty in opening his mouth since ankylosis of the left TMJ recurred. On the initial clinical examination, maximum mouth opening (MMO; maximum interincisal distance) was less than 2 mm (Fig. 1). The panoramic radiograph revealed bone mass of the left mandibular condylar process (Fig. 2a). There were irregular expanded bony contour, cortical thickening, and diffused sclerosis around the left TMJ on three-dimensional computed tomography images (Fig. 2b). Increased uptake in the left TMJ were observed in bone scan (Fig. 2c). These diagnostic images confirmed a true bony ankylosis of the left TMJ, which was assessed as type IV TMJ ankylosis according to Sawhney’s classification [8].

### First procedure, 12 years old; costochondral graft

**Fig. 3:**
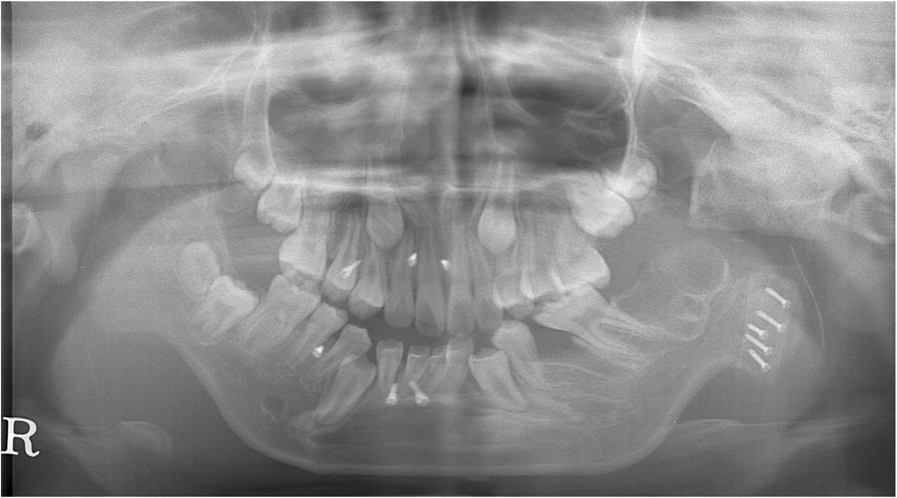
Postoperative panoramic radiograph after surgery. The costochondral graft was adjusted to the left condyle area and secured on the left mandibular ramus by five bicortical screws for rigid internal fixation

Removal of ankylotic mass and TMJ reconstruction with costochondral graft were planned. The left TMJ was approached through a preauricular incision, and excision of ankylotic mass was done. The costochondral graft, harvested from the fifth rib of the right side, was adjusted to the condyle area. It was carefully done so the cartilaginous part of the graft is not separated from the bone. A temporalis muscle fascia flap was rotated over the arch into the joint and was lined in the TMJ space in order to reconstruct the roof of the new glenoid fossa. The deep temporalis fascia and the superficial muscle layer were transferred to construct a barrier, to support the function of the reconstructed ramus/condyle unit and to maintain flap vascularity. Five bicortical screws were secured on the left mandibular ramus for rigid internal fixation of grafted bone (Fig. 3).

**Fig. 4:**
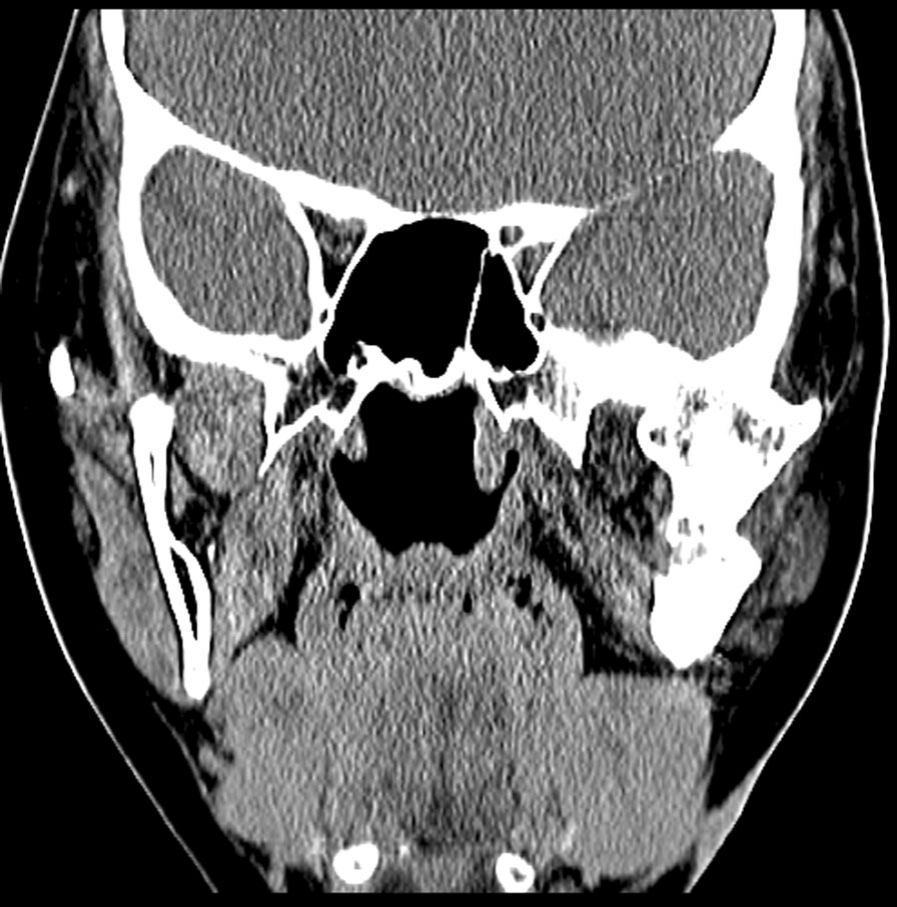
Computed tomography after 1 year postoperatively. Bony ankylosis was confirmed on the left TMJ

A postoperative MMO measured up to 20 mm, and it was increased to 26 mm after 5 weeks postoperatively. However, newly growing bone encapsulated the grafted costochondral head resulting in a limited mouth opening of less than 12 mm within a year postoperatively. Radiographic and clinical evidences confirmed re-ankylosis of the left TMJ. Computed tomography revealed bony ankylosis on the left TMJ (Fig. 4). Increased uptake lesion in the mid-ascending ramus area of the left mandible was also observed in the bone scan.

### Second procedure, 13 years old; gap arthroplasty with interpositional silicone block

**Fig. 5:**
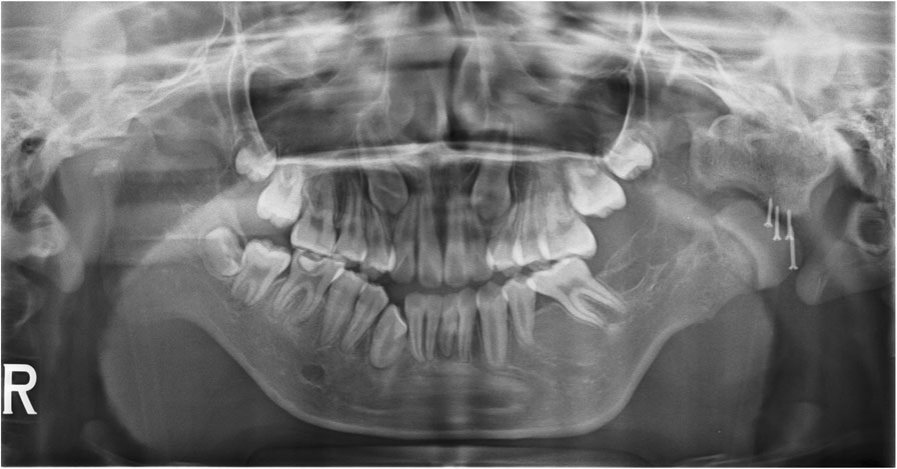
Postoperative panoramic radiograph after surgery. Silicone block with full coverage of the glenoid fossa was placed and fixed in the cranium

Removal of costochondral graft and gap arthroplasty using interpositional silicone block was carried out when the patient was 13 years of age. A 12-mm-thick silicone block with full coverage of the glenoid fossa was placed and fixed in the cranium (Fig. 5). A postoperative MMO measured up to 26 mm in the 3 weeks postoperative follow-up. However, MMO was decreased to 18 mm after 1 year postoperatively. There was also gradual reduction of mouth opening, and MMO was noted to be 12 mm after 1 year and 4 months postoperatively. Radiographic finding also revealed the re-ankylosis of the left TMJ. Therefore, alloplastic temporomandibular joint reconstruction combined with partial mandibulectomy was planned.

### Third procedure, 15 years old; reconstruction with alloplastic condyle

**Fig. 6:**
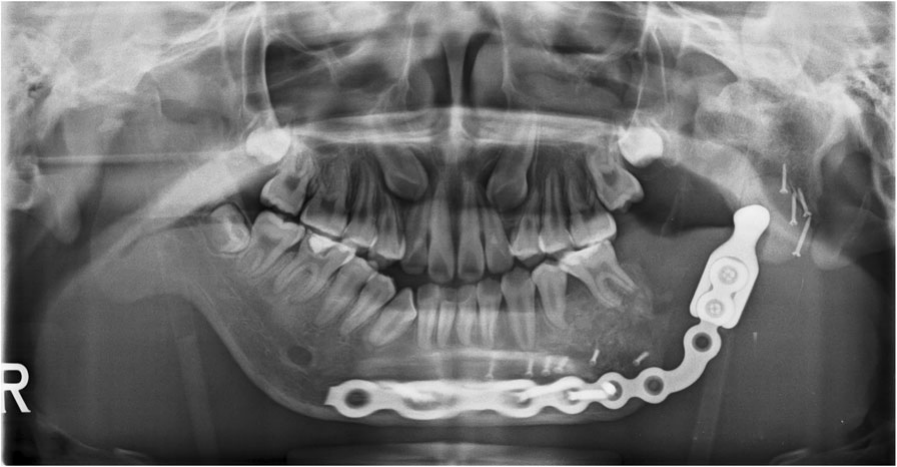
Postoperative panoramic radiograph after surgery. Alloplastic temporomandibular joint reconstruction combined with partial mandibulectomy was performed

Partial mandibulectomy and placement of metallic condylar head prostheses were performed (Fig. 6). After 1 year postoperative follow-up, bone scan revealed the absence of abnormal bone uptake in the reconstructed TMJ area. Mouth opening was consistently measured up to 40 mm without any signs or symptoms of re-ankylosis.

### Fourth procedure, 17 years old; reconstruction of mandibular ramus with iliac bone

**Fig. 7:**
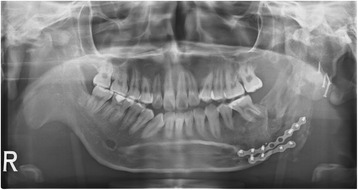
Postoperative panoramic radiograph after surgery. Reconstruction of the mandibular ramus with cortico-cancellous iliac bone graft was performed

**Fig. 8:**
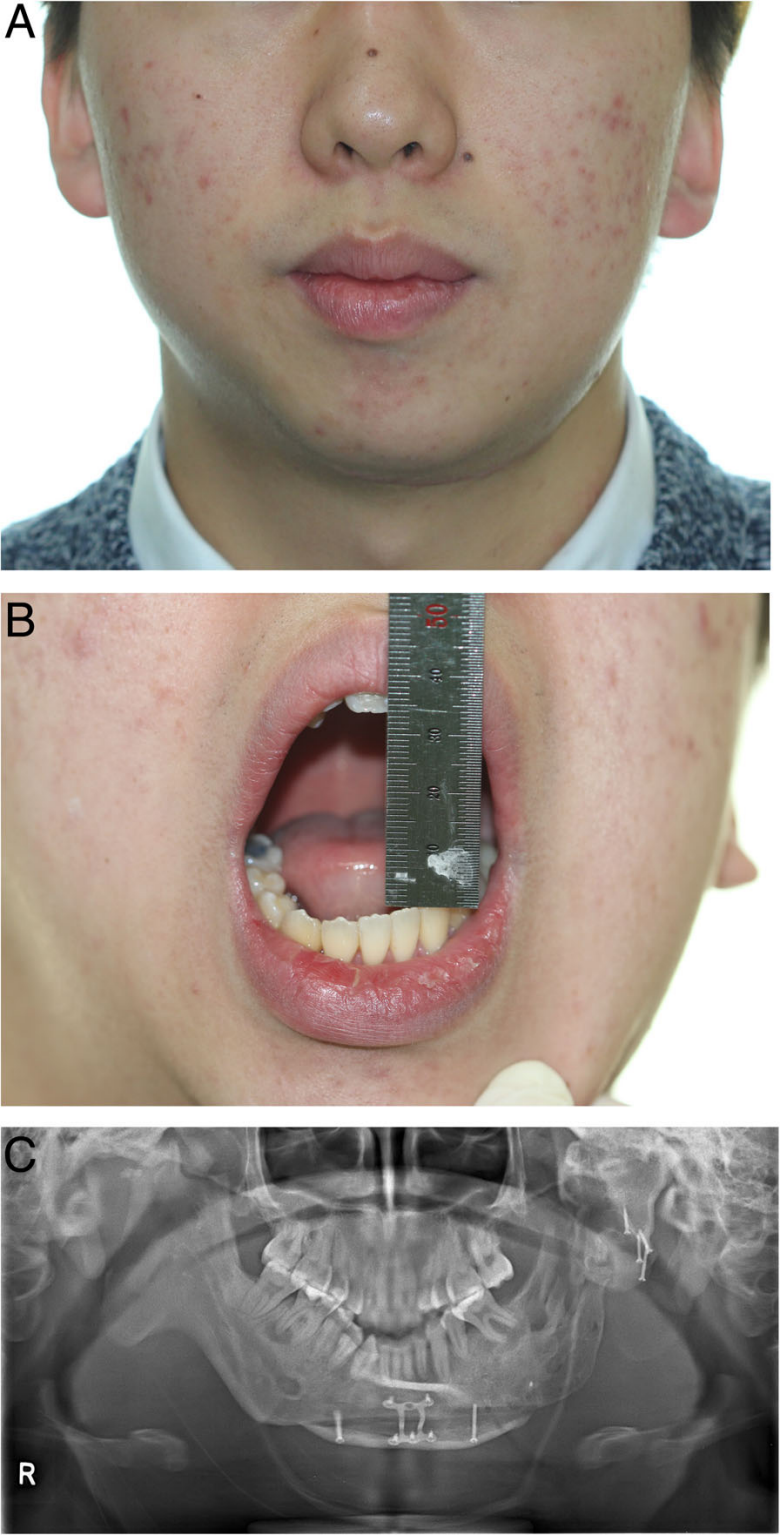
The 3 years of long-term follow-up after last surgery. **a** Frontal view when he was 19 years old. Mandibular asymmetry was not observed. **b** Maximum mouth opening was noted to be 35 mm and **c** the evidence of reankylosis was not observed on panoramic radiograph after 3 years postoperatively

Reconstruction of the mandibular ramus with cortico-cancellous iliac bone graft was performed to compensate additional growth of the mandible after confirming the facial growth completion through a serial cephalometric analysis at 17 years of age (Fig. 7). During clinical examination at 6 months postoperatively, the patient showed a good range of motion with MMO of 35 mm. The patient had a long-term follow-up of orthodontic treatment for occlusal stabilization consistently, and MMO was being maintained at 35 mm during the first 3 years of follow-up (Fig. 8a). Mandibular asymmetry (Fig. 8b) and the evidence of re-ankylosis on radiographic evaluation (Fig. 8c) were not observed.

## Discussion

This case report introduces sequential management of the left TMJ ankylosis resulted from trauma in early childhood. TMJ reconstruction was carried out using costal cartilage graft after removing ankylosed tissues of the left TMJ. The use of costochondral graft is a common practice for condyle reconstruction in children with ankylosis. The advantages of this procedure include biologic and anatomic similarity to the mandibular condyle, growth potential in pediatric patients, ease of harvesting and adapting the graft, and low morbidity of the donor site [7, 9]. Because of the similarities of its primary and secondary cartilages to those of the mandibular condyle [9], the costochondral graft will provide growth potential and keep pace with the growth of the unaffected side, maintaining mandibular symmetry throughout growth [7]. However, long-term studies on mandibular growth in children with reconstructed TMJs using costochondral grafts show excessive growth on the treated side, occurring in 54 % of the 72 cases evaluated, and only 38 % of the cases presented equal growth with the opposite side, and ankylosis can be expected in rare instances from the recipient site [10–12]. It is recommended that early mobilization and aggressive physiotherapy should be done after releasing the intermaxillary fixation (IMF) and immediately postoperatively for patients reconstructed with the costochondral graft [5]. In this case, there were radiographic and clinical evidences confirming re-ankylosis on the recipient site after 1 year postoperatively and mainly due to the IMF with elastic over 8 weeks after surgery and non-compliance with proper physiotherapy.

Even though proper healing was expected after reconstruction of the left TMJ with costal cartilage graft, additional surgical interventions, including interpositional arthroplasty, were performed due to re-ankylosis of the affected site. There is no consensus in the literature on a standard protocol for management of TMJ ankylosis, but three modalities are commonly used: (1) gap arthroplasty, (2) interpositional arthroplasty, and (3) excision and articular reconstruction [13]. The first modality is performed without intervening grafts or materials and is based on resection of ankylosed bone. According to the literature, a minimum of 15-mm gap is recommended between the recontoured glenoid fossa and the mandible for preventing re-ankylosis [14, 15]. Gap arthroplasty offers an advantage of a simple procedure and requires a short surgical time. However, disadvantages include the following: (1) creation of a pseudoarticulation, (2) a short mandibular ramus with anterior open bite in bilateral cases and posterior open bite in unilateral cases, (3) failure of removal of pathologic bone tissue, and (4) high risk of recurrence [16, 17]. The interpositional arthroplasty is recommended after gap arthroplasty as a means to limit resection and recurrence. In this procedure, autogenous and alloplastic materials are placed in the osteotomized area. The important criteria in the choice of graft or interpositional material are cost, esthetic consequences after graft removal, long-term behavior, risk of infection, biocompatibility, tolerance, and prevention of recurrence [16]. In a comparative study, satisfactory results were observed in 92 % of cases with skin graft [18] and 83 % of cases with temporal muscle flaps [13]. Among the several alloplastic materials, gold foil, silastic sheet, acrylic, stainless steel, and silicone prostheses have been used [19–21].

Alloplastic temporomandibular joint replacement can provide a viable option for the multiple operated patients with distorted TMJ anatomy or severe anatomical discrepancies involving the TMJ with recurrent ankylosis [22, 23]. Orthopedic surgeons often prefer alloplastic prosthesis in the replacement of joint in similar situations involving other joints over the use of autogenous bone into the area where reactive or heterotropic bone is forming [24]. In this case, alloplastic prosthesis could be a good selection to prevent recurrent TMJ ankylosis in a growing child.

## Conclusion

It is proposed that alloplastic prosthesis could be performed to prevent TMJ re-ankylosis for growing pediatric patients with TMJ ankylosis in the beginning. And then there is an additional surgery to compensate mandibular growth after confirming growth completion.
